# Psychological Implications of Unemployment Among Bangladesh Civil Service Job Seekers: A Pilot Study

**DOI:** 10.3389/fpsyt.2019.00578

**Published:** 2019-08-12

**Authors:** Md. Abdur Rafi, Mohammed A. Mamun, Kamrul Hsan, Moazzem Hossain, David Gozal

**Affiliations:** ^1^Rajshahi Medical College, Rajshahi, Bangladesh; ^2^Undergraduate Research Organization, Dhaka, Bangladesh; ^3^Department of Public Health & Informatics, Jahangirnagar University, Dhaka, Bangladesh; ^4^Institute of Allergy and Clinical Immunology of Bangladesh, Dhaka, Bangladesh; ^5^Department of Child Health and the Child Health Research Institute, The University of Missouri School of Medicine, Columbia, MO, United States

**Keywords:** depression, anxiety, stress, job seekers, unemployed graduates, Bangladesh Civil Service

## Abstract

**Background:** Recent trends suggest that university graduates seeking jobs are more susceptible to common mental disorders, such as depression, anxiety, or stress. However, the mental health issues among unemployed graduates has not been explored in Bangladesh yet.

**Aims:** This study aimed to assess for the first time the prevalence and associated risk factors of depression, anxiety, and stress among Bangladesh Civil Service (BCS) job seekers. Three hundred four graduates residing in Rajshahi, Bangladesh, who were preparing to attend the 40^th^ BCS examination, the most sought-after employment opportunity in the country, were surveyed.

**Methods:** Measures included socio-demographics, field of study, and career-related variables, and the Bangla Depression Anxiety Stress Scale (DASS-21). Chi-square test, Fisher exact test, and binary logistic regression with “depression,” “anxiety,” and “stress” as the dependent variables were carried out to identify the factors associated with these.

**Results:** Overall, the prevalence of moderate to extremely severe depression, anxiety, and stress was 49.3%, 53.6%, and 28.3%, respectively, with no detectable differences between genders. Insecurity related to a BCS job (OR = 0.41; CI = 0.26–0.65, *p* < 0.001; ref: job insecurity), family and social pressure to obtain a BCS job (OR = 4.58; CI = 1.67–12.56, *p* < 0.001), and stress (OR = 8.33; CI = 4.47–15.51, *p* < 0.001) emerged as independent predictors for depression. In addition, having part-time job was associated with anxiety (OR = 2.38; CI = 1.34–4.23, *p* = 0.003), and security in a BCS job and serving the nation through this job were negatively associated with stress (OR = 0.59; CI = 0.35–0.98, *p* = 0.042 vs. OR = 0.59; CI = 0.36–1.00, *p* = 0.05).

**Conclusion:** The relatively high rates of depression, anxiety, and stress among graduate job seekers should prompt implementation of market force initiatives that incorporate interventions related to the major risk factors uncovered herein.

## Introduction

Achieving a university degree is nowadays considered an important key to professional and societal success. The number of university students has markedly increased over the last half century along with remarkable expansion of universities and their campuses, reflecting the high demand. The increased numbers of university graduates are then anticipated to enter the labor force, but since the job market is not expanding fast enough to cope with the increasing number of graduates, increased competition and job uncertainty have emerged (1). Consequently, the number of unemployed and underemployed university graduates has steadily increased, with additional new graduates entering the job market and the inability of governments and the private sector to provide appropriate employment solutions (2). The rising unemployment or underemployment is likely to impose adverse societal and personal consequences that are not necessarily appropriately addressed. Indeed, recent studies have shown that the situational factors driving either unemployment or underemployment (UUE) were quite diverse, and appeared to be associated with the risk of manifesting common mental health consequences, such as depression, anxiety, and stress (2–7). Moreover, these common mental health problems account for at least 90% of suicide cases. Indeed, such events have been linked to but not restricted to a large spectrum of psychological and psychiatric disorders encompassing conditions such as depression disorder, adjustment disorders, alcohol and drug dependency and addiction, anxiety disorders, schizophrenia and other psychoses, and sleep disorders to name a few (8–11). In the sense of unemployment, from recent review articles, it was suggested as an unmet factor for promoting the vulnerability of suicidal behaviors by adding to the impact of stressful life events and also of suicide by evaluating proximal suicide risk factors (i.e., mental illness, family conflict, dissatisfaction, etc.) (12–14).

UUE-related elements such as economic distress and joblessness, as well as job insecurity, frequently underlie feelings of failure, which in turn may lead to depression, stress, and anxiety (3–6). Moreover, the family and social pressures associated with job-seeking activities and higher expectations from university graduates also act as potential mediators of depression and stress disorders among university graduates exploring the job markets (2). In parallel, socio-demographic characteristics (i.e., female, unmarried, lower quality of life, family socioeconomic status, academic major, educational expense loan, and willingness to accept irregular employment) are also significantly associated with the risk for mental disturbances (3, 15).

In Bangladesh, the situation is remarkably comparable to current global trends. According to a report of the Bangladesh Bureau of Statistics (BBS), about 11.2% of youths with tertiary education were unemployed in the 2016–2017 economic year. This unemployment rate is nearly threefold higher than the national unemployment rate of 4.2% (16). At least 6.6 million Bangladeshi university graduates, who are *a priori* qualified for permanent jobs, are unable to secure an employment opportunity as the job market is not expanding fast enough to cope with the increasing number of graduates (1). Moreover, the socio-cultural structure in Bangladesh has evolved in such a way that university graduates will preferentially seek to become government employees rather than to become entrepreneurs. Accordingly, each year, an ever-increasing number of graduates will try to obtain employment in the Bangladesh Civil Service (BCS), the most prestigious and sought-after governmental employer in Bangladesh, and pursue available cadre positions (note: a job holder in the BCS is referred to as a cadre). For example, a total of 346,440 candidates applied for 2,024 vacant positions during the 38^th^ BCS examination (i.e., 171 candidates per position) (17). Therefore, the vast majority of university graduates will falter and be unable to secure their aspirational job. Even in the context of multiple repeated year-after-year attempts, this can have consequent adverse effects on productivity as well as potentially promote reduced self-esteem or more serious psychiatric disorders.

In these contextual settings, it has become apparent that the global prevalence of common mental disorders is rising, especially in middle- and low-income countries, and leads to considerable losses in health and productivity (18). A recent systematic review focused on Bangladesh showed that the prevalence of common mental health problems has been reported in a very wide range, from 6.5% to 31.0% among adults and from 13.4% to 22.9% among children (19).

We are unaware of any previous studies examining potential issues of mental health among university graduates seeking employment. Recent reports in the news of suicides associated with unemployment among educated individuals residing inside (1, 20) or outside (12–14) of Bangladesh prompted us to carry out the present study which aimed to evaluate the prevalence of depression, anxiety, and stress among university graduate BCS job seekers, and potentially identify risk factors associated with such mental health issues.

## Methods

### Participants and Procedure

The BCS examination is the most competitive job-related examination in Bangladesh, which aims to select civil servants and is run by the Bangladesh Public Service Commission. The recruited officers are called BCS cadres, where 27 types of cadres (including general, technical, and mixed) are defined in the context of the Civil Service of Bangladesh (21). The current cross-sectional pilot study was conducted among university graduates living in Rajshahi, Bangladesh, who were preparing themselves to participate in the 40^th^ BCS examination to become a cadre. The survey was conducted between August and October 2018. A total of 350 printed questionnaires were distributed among students at three randomly selected BCS preparation coaching centers. The questionnaire took about 30 minutes to complete and data from 315 participants were collected (response rate 90%). After removal of incomplete questionnaires (n = 9), 304 were retained for final analyses.

### Ethics

This study was approved by the Institutional Review Board of the Institute of Allergy and Clinical Immunology of Bangladesh (IACIB), Savar, Dhaka, Bangladesh [ref. no: IRBIACIB/CEC/07201801]. Verbal and formal written consent from all respondents were obtained before participating in the study. All subjects were also informed about the i) nature and purpose of the study, ii) procedure of the study, iii) right to refuse, and iv) right to withdraw from participating in the study. The participants did not gain any financial benefit from taking part in the study. Confidentiality of data and anonymity to the participants were ensured.

### Measures

#### Socio-Demographics

Socio-demographics included age, sex, residence (city or village), relationship status (i.e., single, engaged in relationship, or married), and socioeconomic status. Socioeconomic status was appraised from monthly total family income, which was categorized into higher, middle, and lower income groups corresponding to annual income of > BDT30000, BDT15000 to BDT30000, and < BDT15000, respectively.

#### Study and Job-Related Questions

Study and career-related questions included the type of higher learning institution (public university or colleges under national university), year of graduation, whether they were doing any part-time job (“yes” or “no” response), whether they attended the BCS examination before or not, cadre of choice, and factors behind choosing the BCS as a professional career. For accessing driving factors of BCS preference, multiple-choice responses were offered while covering probable options (e.g., security of the job, high salary, family and social expectation, to serve the nation). Regarding BCS cadre choice, multiple options were included (e.g., administrative, education, police).

#### Bangla Depression Anxiety Stress Scale (DASS)

This is a 21-item self-report inventory that provides scores on three subscales, i.e., depression (7 items), anxiety (7 items), and stress (7 items) (22). Each item was rated on a four-point Likert scale ranging from “never” (0) to “always” (3). In the present study, the Bangla version DASS-21 was used (23). Moderate, severe, and very severe were combined to denote a problematic score for depression, anxiety, and stress in this study. In the Bangla version, Cronbach’s alpha for the depression, anxiety, and stress subscales were 0.99, 0.96, and 0.96, respectively (23). For this study the Cronbach’s alpha for the depression, anxiety, and stress subscales were 0.787, 0.783, and 0.834, respectively, and the overall Cronbach’s α was 0.919.

### Statistical Analysis

Statistical Package for Social Science (SPSS) version 22.0 was used for data analysis. Continuous variables, i.e., age, are not shown in the tables, but categorical variables are presented as descriptive statistics (e.g., frequencies, percentages, and Chi-square and Fisher exact test). All variables were entered into binary logistic regression with “depression,” “anxiety,” and “stress” as the dependent variables. The results of logistic regression were interpreted with 95% confidence intervals.

## Results

A total of 304 BCS candidates (mean age 24.2 ± 1.6 years; females: 170 (55.9%)) participated in the study. The majority of the respondents (78.3%) were from rural areas and belonged to the middle socioeconomic group (59.9%). The vast majority were single, with 14.1% being married and 16.4% involved in a relationship. About 57.6% of participants had graduated from colleges compared to 42.7% having graduated from a university. In addition, 77.3% were exclusively preparing for the BCS examination without taking on any part-time job, and for the majority (82.2%), this was their first attempt to take the BCS examination (**Table 1**).

**Table 1:** Distribution of variables among respondents by depression, anxiety, and stress.

Variables	Total = 304 n (%)	Depression				Anxiety				Stress			
		Yes (%)	*X* *^2^* value	df	*p*-value	Yes (%)	*X* *^2^* value	df	*p*-value	Yes (%)	*X* *^2^* value	df	*p*-value

**Socio-demographics**													
**Gender**													
Male	134 (44.1)	59 (44.0)	2.705	1	0.100	68 (50.7)	0.795	1	0.373	33 (24.6)	1.585	1	0.208
Female	170 (55.9)	91 (53.5)				95 (55.9)				53 (31.2)			
**Permanent residence**													
Village area	238 (78.3)	115 (48.3)	0.218	1	0.641	132 (55.5)	2.133	1	0.144	69 (29.0)	0.108	1	0.742
City area	56 (18.4)	29 (51.8)				25 (44.6)				15 (26.8)			
**Relationship status**													
Married	43 (14.1)	20 (46.5)	0.283	2	0.868	22 (51.2)	1.049	2	0.592	13 (30.2)	2.071	2	0.355
In a relationship	50 (16.4)	26 (52.0)				30 (60.0)				10 (20.0)			
Single	210 (69.1)	103 (49.0)				110 (52.4)				63 (30.0)			
**Socioeconomic status**													
Lower class	51 (16.8)	29 (56.9)	1.458	2	0.482	27 (52.9)	1.169	2	0.558	19 (37.3)	5.035	2	0.081
Middle class	182 (59.9)	88 (48.4)				94 (51.6)				43 (13.6)			
Upper class	71 (23.4)	33 (46.5)				42 (59.2)				24 (33.8)			
**Study- and job-related questions**													
**Graduation institute**													
College	175 (57.6)	85 (48.6)	0.098	1	0.754	95 (54.3)	0.074	1	0.786	52 (29.7)	0.413	1	0.521
University	129 (42.4)	65 (50.4)				68 (52.7)				34 (26.4)			
**Graduation year**													
2015 to 2012	52 (17.1)	30 (57.7)	5.173	3	0.160	29 (55.8)	2.441	3	0.486	20 (38.5)	4.227	3	0.238
2016	52 (17.1)	19 (36.5)				23 (44.2)				14 (26.9)			
2017	84 (27.6)	44 (52.4)				45 (53.6)				25 (29.8)			
2018	116 (38.2)	57 (49.1)				66 (56.9)				27 (23.3)			
**Current part-time job**													
Yes	69 (22.7)	31 (44.9)	0.696	1	0.404	48 (69.6)	9.127	1	0.003	21 (30.4)	0.203	1	0.653
No	235 (77.3)	119 (50.6)				115 (48.9)				65 (27.7)			
**Previous BCS attempt**													
Yes	54 (17.8)	25 (46.3)	0.244	1	0.622	28 (51.9)	0.082	1	0.774	16 (29.6)	0.058	1	0.809
No	250 (82.2)	125 (50.0)				135 (54.)				70 (28.0)			
**BCS completed stage**													
Written stage	5 (1.6)	2 (40.0)	0.242	2	0.886	3 (60.0)	0.115	2	0.944	2 (40.0)	0.361	2	0.835
Preliminary stage	44 (14.5)	21 (47.7)				23 (52.3)				12 (27.3)			
None	255	127 (49.8)				137 (53.7)				86 (28.3)			
Reasons for BCS preference*													
Security of job	145 (47.7)	55 (37.9)	14.442	1	0.001	72 (49.7)	1.751	1	0.186	33 (22.8)	4.180	1	0.041
High salary	143 (47.0)	70 (49.0)	0.017	1	0.898	75 (52.4)	0.149	1	0.700	35 (24.5)	1.936	1	0.164
More scope of practicing power	105 (34.5)	56 (53.3)	1.022	1	0.313	57 (54.3)	0.029	1	0.865	27 (25.7)	0.524	1	0.469
Easier working environment	89 (29.3)	45 (50.6)	0.75	1	0.784	50 (56.2)	0.332	1	0.564	22 (24.7)	0.791	1	0.374
Family and social pressure	25 (8.2)	20 (80.0)	10.243	1	0.001	15 (60.0)	0.446	1	0.504	8 (32.0)	0.185	1	0.667
To serve the nation	133 (43.8)	61 (45.9)	1.144	1	0.285	70 (53.6)	0.093	1	0.761	30 (22.6)	3.831	1	0.050
**Preferred cadre of BCS**													
Administration	168 (55.3)	85 (50.6)	3.158	3	0.368	92 (54.8)	3.134	3	0.371	47 (28.0)	1.960	3	0.581
Education	103 (33.9)	45 (43.7)				58 (56.3)				27 (16.2)			
Police	22 (7.2)	13 (59.1)				9 (40.9)				7 (31.8)			
Others	11 (3.6)	7 (63.6)				4 (36.4)				5 (45.5)			
**Depression, anxiety, and stress**													
**Depression status**													
No	154 (50.7)	–	–	–	–	80 (51.9)	0.350	1	0.554	15 (9.7)	52.935	1	0.000
Yes	150 (49.3)	–	–	–	–	83 (55.3)				71 (47.3)			
**Anxiety status**													
No	141 (46.4)	67 (47.5)	0.350	1	0.554	–	–	–	–	37 (26.2)	0.544	1	0.461
Yes	163 (53.6)	83 (27.3)				–	–	–	–	49 (30.1)			
**Stress status**													
No	218 (71.7)	79 (36.2)	52.935	1	0.000	114 (52.3)	0.544	1	0.461	–	–	–	–
Yes	86 (28.3)	71 (82.6)				49 (57.0)				–	–	–	–

*Multiple response.

The overall prevalence of depression, anxiety, and stress among the participants was 49.3%, 53.6%, and 28.3% respectively, with women more likely to report these symptoms compared to men, albeit without reaching statistical significance [prevalence of depression, anxiety, and stress was (53.5% vs. 44.0%; *X* *^2^* = 2.70, df = 1, *p* = 0.100), (55.9% vs. 50.7%;* X* *^2^* = 0.79, df = 1, *p* = 0.373), and (31.2% vs. 24.6%; *X* *^2^* = 1.58, df = 1, *p* = 0.208), respectively] (**Table 1**).

Participants doing any part-time job were more likely to suffer from anxiety (69.6%; *X* *^2^* = 9.13, df = 1, *p* = 0.003). Moreover, the prevalence of depression was lower among the candidates who had chosen the BCS as a career for better job security (37.9%; *X* *^2^* = 14.44, df = 1, *p* < 0.001). In contrast, when the reason for choosing BCS was family and social pressure, the risk of experiencing depressive disorder was increased (80%; *X* *^2^* = 10.24, df = 1, *p* < 0.001). Moreover, those who had chosen the career to serve the nation were less likely to report stress (22.6%; *X* *^2^* = 3.83, df = 1, *p* = 0.05). Depression and stress were significantly correlated (*p* = 0.000), but no association was found between depression and anxiety, and between anxiety and stress. (**Table 1**)

Predictors of depression, anxiety, and stress from the multivariate logistic regression analysis are shown in **Table 2**. Seeking BCS employment for family and social pressure reasons (OR = 4.58; CI = 1.67–12.56, *p* < 0.001) and suffering from stress (OR = 8.33; CI = 4.47–15.51, *p* < 0.001) emerged as independent risk factors of depression, whereas seeking BCS employment as reflecting a secure job was negatively associated with depression (OR = 0.41; CI = 0.26–0.65, *p* < 0.001). Regarding anxiety risk, engaging in a current part-time job (OR = 2.38; CI = 1.34–4.23, *p* = 0.003) emerged as an independent risk factor. Moreover, BCS for security of job and to serve the nation were negatively associated with stress (OR = 0.59; CI = 0.35–0.98, *p* = 0.042 vs. OR = 0.59; CI = 0.36–1.00, *p* = 0.05) (**Table 2**).

**Table 2:** Binary regression analysis of the variables by depression, anxiety, and stress.

Variables	Depression			Anxiety			Stress		
	OR	95% CI	p-value	OR	95% CI	p-value	OR	95% CI	p-value
**Gender**									
Female	1.464	(0.929–2.308)	0.101	1.229	(0.781–1.936)	0.373	1.386	(0.833–2.308)	0.209
Male	Reference			Reference			Reference		
**Permanent residence**									
Village area	0.870	(0.486–1.559)	0.641	1.544	(0.860–2.773)	0.146	1.116	(0.580–2.147)	0.742
City area	Reference			Reference			Reference		
**Relationship status**									
Married	0.903	(0.468–1.743)	0.868	0.952	(0.494–1.836)	0.593	1.001	(0.495–2.006)	0.361
In a relationship	1.125	(0.607–2.086)		1.364	(0.728–2.553)		0.583	(0.275–1.239)	
Single	Reference			Reference			Reference		
**Socioeconomic status**									
Lower class	1.518	(0.736–3.132)	0.485	0.777	(0.376–1.605)	0.559	1.163	(0.549–2.464)	0.083
Middle class	1.078	(0.622–1.868)		0.738	(0.423–1.285)		0.606	(0.333–1.103)	
Upper class	Reference			Reference			Reference		
**Graduation institute**									
College	0.930	(0.590–1.466)	0.754	1.065	(0.675–1.681)	0.786	1.181	(0.710–1.964)	0.521
University	Reference			Reference			Reference		
**Graduation year**									
2015 to 2012	1.411	(0.730–2.730)	0.166	0.955	(0.494–1.847)	0.490	2.060	(1.018–4.171)	0.244
2016	0.596	(0.304–1.167)		0.601	(0.311–1.162)		1.214	(0.574–2.568)	
2017	1.139	(0.649–1.997)		0.874	(0.497–1.538)		1.397	(0.740–2.638)	
2018	Reference			Reference			Reference		
**Current part-time job**									
Yes	0.795	(0.464–1.363)	0.405	2.385	(1.345–4.230)	0.003	1.144	(0.636–2.058)	0.653
No	Reference			Reference			Reference		
**Previous BCS attempt**									
Yes	0.862	(0.478–1.554)	0.622	0.917	(0.509–1.653)	0.774	1.083	(0.567–2.066)	0.810
No	Reference			Reference			Reference		
**BCS completed stage**									
Written stage	0.672	(0.110–4.089)	0.887	1.292	(0.212–7.863)	0.944	1.694	(0.277–10.352)	0.838
Preliminary stage	0.920	(.485–1.746)		0.943	(0.497–1.790)		0.953	(0.465–1.953)	
None	Reference			Reference			Reference		
**Reasons for BCS preference**									
Security of job	0.412	(0.260–0.653)	<0.001	0.737	(0.469–1.159)	0.186	0.589	(0.354–0.981)	0.042
High salary	0.971	(0.619–1.523)	0.898	0.915	(0.582–1.437)	0.700	0.699	(0.422–1.159)	0.165
More scope of practicing power	1.277	(0.795–2.050)	0.312	1.042	(0.648–1.674)	0.865	0.821	(0.482–1.400)	0.469
Easier working environment	1.071	(0.654–1.756)	0.784	1.157	(0.704–1.902)	0.565	0.775	(0.441–1.361)	0.375
Family & social pressure	4.585	(1.673–12.560)	<0.001	1.328	(0.577–3.057)	0.505	1.213	(0.503–2.924)	0.668
To serve the nation	0.781	(0.496–1.229)	0.285	0.932	(0.592–1.468)	0.761	0.598	(0.357–1.003)	0.050
**Preferred cadre of BCS**									
Administration	0.585	(0.165–2.074)	0.374	2.118	(0.598–7.509)	0.384	0.466	(0.136–1.601)	0.596
Education	0.443	(0.122–1.608)		2.256	(0.622–8.183)		0.426	(0.120–1.511)	
Police	0.801	(0.185–3.676)		1.212	(0.272–5.396)		0.560	(0.126–2.479)	
Others	Reference			Reference			Reference		
**Depression status**									
Yes	–	–	–	1.146	(0.730–1.799)	0.554	8.328	(4.473–15.507)	<0.001
No	–	–	–	Reference			Reference		
**Anxiety status**									
Yes	1.146	(0.730–1.799)	0.554	–	–	–	1.208	(0.731–1.998)	0.461
No	Reference			–	–	–	Reference		
**Stress status**									
Yes	8.328	(4.473–15.507)	<0.001	1.208	(0.731–1.998)	0.461	–	–	–
No	Reference			Reference			–	–	–

## Discussion

Unemployment among university graduates is one of the major social problems in Bangladesh, and the unemployment rates have continued to increase, most likely due to insufficient job creation despite a consistently high economic growth over the last several years (1, 16). Considering that the mental health issues among unemployed graduates has not been explored in Bangladesh previously (24–32), the present study aimed at addressing the gap in potential links between depression, anxiety, and stress, and unemployment in this highly educated sector of the population.

High rates of depression, anxiety, and stress were detected in this cohort. Unfortunately, since there are no other studies among job-seeking university graduates in Bangladesh, more rigorous comparisons to elucidate temporal trends or additional contributing factors are precluded. The only study conducted among medical students in Bangladesh using the same instrument also showed high prevalence rates of depression, anxiety, and stress (54.3%, 64.8%, and 59.0% respectively) (33). The elevated prevalence of these issues among medical students may be explained by the significant academic, psychosocial, and existential stressors for coping imposed by the medical college academic curriculum and learning schedules (33, 34). In this setting, the prevalence rates reported from other countries around the world have revealed substantial variability suggesting that both local social and cultural factors, as well as the underlying common elements promoting the emergence of depression, anxiety, and stress, may lead to marked heterogeneity in the prevalence rates of these psychiatric disturbances. Notwithstanding, higher prevalence of these psychiatric symptoms is universally more likely to occur among unemployed graduates all over the world (2–6). Previous studies have reported elevated rates of depression, anxiety, and stress among involuntarily unemployed individuals. For example, the following rates were estimated in the US: depression [D] = 29%, anxiety [A] = 31%, and stress [S] = 28% (35). Similarly, among unemployed adults after the economic crisis in Greece: D = 32.2%; A = 39.7%, and S = 33% (15); in Spain: D = 51.5% and A = 35.5% (36); among unemployed university graduates in Korea: D = 39.5% (3); among unemployed graduates in the UK: S = 69.4% (2); and finally, among unemployed people in Denmark: S = 10.4% (6). Moreover, studies conducted in neighboring countries using DASS-21 showed that the prevalence of these problems was also high among college students in Pakistan, D = 35.9%, A = 64%, and S = 38.5% (37); among medical students in India, D = 32.0%, A = 40.1%, and S = 43.8% (38); and among medical students in Nepal: D = 29.9%, A = 41.1%, and S = 27% (39). Additionally, across the world, the rate varies, with a wide range among university students [such as D = 37.2%, A = 63%, and S = 23.7% in Malaysia (40); D = 27.1%, A = 47.1%, and S = 27% in Turkey (41); and D = 23%, A = 25%, and S = 26% in the United States (42)]. (Please see **Online Supplemental Table**.)

In general, women are more likely to experience job-seeking stress and depression compared to men (7). Moreover, when women were also single, the latter seemed to operate as a mediator of depression (15), while conversely, being married appeared to dampen the likelihood of depression among unemployed women while imposing the reverse effect in men (7). Of note, we found no significant associations between mental health symptoms and gender, even when incorporating marital relationship status in the analyses.

Lower-socioeconomic-group participants are also more likely to experience mental health symptoms when compared to higher socioeconomic groups (32, 43). Thus, economic distress along with family and social pressure for job attainment fosters the emergence of mental health problems (2, 4–6). Younger job seekers who are willing to accept irregular employment (3) or have chosen to take a stopgap job that does not match their qualifications and skill level experienced greater psychiatric problems (44). In a study among Bangladeshi garment workers, part-time employment was found to be associated with depressive disorders (32). In the present study, participants who were engaged in a part-time job were at higher risk of reporting symptoms of anxiety. This may possibly due to the pressure of family poverty fostering economic uncertainty and associated anxiety.

Moreover, achievement motivation may play also play a role in anxiety, since unemployed individuals may be retain increased hope of securing the desired job, while the underemployed may have partially given up hope and accepted failure as their reality (2). However, repeated previous attempts in the BCS examination and failure at the higher stages (mentioned hierarchically as viva, written, and preliminary stage) did not seem to exert any role in the occurrence of mental disturbances compared to those who had never taken the exam. Thus, sustained joblessness and failure of getting a job would be anticipated to foster mental impairments and decrease well-being and quality of life, ultimately leading to depression, anxiety, and stress (3, 4).

There is consistent evidence that losing job security, which can play a role in UUE at any time, will impose significant adverse effects on psychological co-morbidity and disturbances (2, 4, 45). Therefore, study participants who were insecure about the BCS job were more likely to suffer from depression and stress. Moreover, participants reporting family and social pressures to become a BCS cadre were more likely to suffer from depression, as perceived social pressure and stress accelerate the onset of depressive disorders (2). Conversely, social support acts as an important factor to decrease job-searching stress as well as to prevent depression (3). Interestingly, choosing BCS jobs with the aim of serving the nation reflected as a negative predictor of stress. More altruistic goals and potentially less socioeconomic pressure may underlie this observation.

## Limitations of the Study

There are several limitations to this study that deserve mention. Firstly, our study was cross-sectional in nature, and therefore, a stable and potentially causal relationship between depression, anxiety, stress, and their socio-demographic correlates cannot be ascertained. The findings are also limited by the self-reported questionnaire responses, which might be influenced in the context of method bias, memory recall biases, and social desirability biases. Such biases can be potentially removed in future studies through implementation of different methodologies. The relatively small sample size should also be viewed as a potential limitation. Furthermore, the present study was conducted exclusively in the Rajshahi region of the country, such that generalizability may not necessarily be possible.

## Implications of the Study

This is the first study in Bangladesh focusing on common mental health problems and university graduate unemployment. Similar to other studies around the world, a large portion of young people are struggling to cope with a new stage of their professional life after completion of their studies and are at high risk of suffering from various psychiatric distresses, like depression, anxiety, and stress (3, 5, 7), whereas securing a suitable and desirable job has significant benefits for mental well-being (2). We should emphasize that the presence of mental disturbances can affect both productivity and prosperity in life and even increase the risk of suicide and suicidal behaviors (12–14). Therefore, special attention to the mental health of unemployed graduates appears mandatory and is predicated on thorough evaluation of predictive risk factors, which will have to be further explored by more expansive and longitudinal interventional studies. Technical, polytechnic, and vocational education venues can also play a crucial role in reducing unemployment by training graduates and secure them employment aligned with their training through better matching of education and job markets. Furthermore, in the setting of high demands for technically and vocationally trained graduates, job opportunities can be leveraged not only within the country but also abroad, a situation that was adopted in China and recently reported (1).

## Conclusions

The present study reports on the high prevalence and associated risk factors of depression, anxiety, and stress among BCS job seekers. The presence of job insecurity and family and social pressures to secure a BCS job are strong predictors for depression, while being already involved in a part-time job emerged as a risk factor for anxiety. In contrast, intent of obtaining a BCS position to serve the nation predicted reduced stress. This study indicates that preventive workforce initiatives aimed at better alignment between educational pipelines and job markets are needed. Furthermore, early mental support and resilience training programs during higher education are needed to potentially mitigate the elevated risk of mental issues among university graduates in Bangladesh by further exploring with a national large-scale sample concerning our yielded risk issues.

## Data Availability

The datasets generated for this study are available on request to the corresponding author.

## Ethics Statement

This study was carried out in accordance with the recommendations of “name of guidelines, name of committee” with written informed consent from all subjects. All subjects gave written informed consent in accordance with the Declaration of Helsinki. The protocol was approved by the Institutional Review Board of the Institute of Allergy and Clinical Immunology of Bangladesh (IACIB), Savar, Dhaka, Bangladesh (Ref. No: IRBIACIB/CEC/07201801).

## Author Contributions

MAR, MM, KH, and MH conceptualized the initial phases of the study, collected and analyzed data, and drafted initial versions of the manuscript. DG contributed to analyses and performed editing of the manuscript for content and style. All authors have approved the final version of the manuscript.

## Funding

DG is supported by National Institutes of Health grants 1R01HL130984 and R56 HL140548.

## Conflict of Interest Statement

The authors declare that the research was conducted in the absence of any commercial or financial relationships that could be construed as a potential conflict of interest.

## Abbreviations

BCS, Bangladesh Civil Service; UUE, Unemployment or underemployment; DASS, Depression Anxiety Stress Scale.
