# Supplementary material for: Psychological Implications of Unemployment Among Bangladesh Civil Service Job Seekers: A Pilot Study
Source: Front Psychiatry. 2019 Aug 12;10:578. doi: 10.3389/fpsyt.2019.00578 (PMC6698720; doi:10.3389/fpsyt.2019.00578)
Supplement: Supplementary file 1 [file Table_1.docx]

**Online Supplemental Table: Comparison of Depression, Anxiety and Stress prevalence rates across different studies.**

| **Authors** | **Study location** | **Study population** | **Assessment tool** | **Main findings** |
| --- | --- | --- | --- | --- |
| Kokaliari, (2018) (12) | Greece | 901 unemployed adults after the economic crisis | Depression Anxiety Stress Scale (DASS-42) | Depression [D]=32.2%; Anxiety [A]=39.7%,;  Stress [S]=33% |
| Howe et al., (2012) (30) | US | 426 involuntary unemployed people | Center for Epidemiological Study Depression Scale & Brief Social Phobia Scale | D=29%; A= 31% |
| Navarro-Abal et al., (2018) (31) | Spain | 244 unemployed people | Zung’s self-rating depression scale & Beck’s Anxiety Inventory | D=51.5%; A=35.5% |
| Lim et al., (2018) (3) | Korea | 124 unemployed university graduates | Korean Beck Depression Inventory-II | D=39.5% |
| Cassidy & Wright, (2008) (2) | UK | 248 unemployed graduates | General Health Questionnaire-12 | S=69.4% |
| Mæhlisen et al., (2018) (6) | Denmark | 35,700 unemployed people | Cohen’s Perceived Stress Scale | S=10.4% |
| Alim, Rabbani, et al., (2017) (28) | Bangladesh | 105 medical students | Bangla DASS-21 | D=54.3%; A=64.8%; S=59.0% |
| Nadeem, Ali, & Buzdar, (2017) (32) | Pakistan | 723 college students | DASS-21 | D=35.9%; A=64%; S=38.5% |
| Taneja, Sachdeva, & Dwivedi, (2018) (33) | India | 187 medical students | DASS-21 | D=32.0%; A=40.1%,; S=43.8% |
| Kunwar, Risal, & Koirala, (2016) (34) | Nepal | 538 medical students | DASS-42 | D=29.9%; A=41.1%; S=27% |
| Shamsuddin et al., (2013) (35) | Malasiaya | 506 university students | DASS-21 | D=37.2%; A=63%; S=23.7% |
| Bayram & Bilgel, (2008) (36) | Turkey | 1,617 university students | DASS-42 | D=27.1%, A=47.1%; S=27 |
| Beiter et al., (2015) (37) | US | 374 undergraduate students | DASS-21 | D=23%; A=25%; S=26% |
